# Supplementary material for: Patterns of Intron Gain and Loss in Fungi
Source: PLoS Biol. 2004 Nov 30;2(12):e422. doi: 10.1371/journal.pbio.0020422 (PMC532390; doi:10.1371/journal.pbio.0020422)
Supplement: Table S1 — Also available at http://genes.mit.edu/NielsenEtAl/. (4.3 MB ZIP). [file pbio.0020422.st001.zip › NielsenEtAl/html/527.html]

AN2093.1.NCU09220.1.MG05755.1.FG07083.1


```
 CLUSTAL W (1.82) Multiple Sequence Alignments - Introns Inserted


Sequence 1: NCU09220.1	678 aa
Sequence 2: MG05755.1	630 aa
Sequence 3: FG07083.1	587 aa
Sequence 4: AN2093.1	614 aa
Alignment Length: 716 aa
Number Identitical Residues: 222 aa
Alignment Score (without introns) 11987


MG05755.1 	MAQEDHAPDSADSAAVTATDSPDPATS---------------TPTPPPPPLPPRPRQPTS
NCU09220.1	MDRDTEAGSNDGTNDNNDTTIPPHTPE---------------SNIEAVDHVPPVPRRPSP
FG07083.1 	MDSDSIN--------------PKPISS---------------VDEDYRPPLPPRP----S
AN2093.1  	MDTKDSNSSQALAIDGGAQTRPTDDHASLTKRLTQPRSDRSQSPEGRPPPLPPRPETLIL
          	*  .    ..  :        *     : :.  :.. :. :.        :** *     

MG05755.1 	PGSTETSK------LQSGATTAITSIDIQTLSFPDGSRGTFSTPGTRAVSTPGRT-----
NCU09220.1	KLSDDGASGALHVAAASRPTTQVSTIDISTLSFPDGSRGTFSTSATSATSATSATRSVAS
FG07083.1 	TNATDPGS------LQGQATTAITPVEIQTLSFPDGSRGTFSTPGPDSIPSPAESGG---
AN2093.1  	LEDGGAAPG-TPRPNVSAVHPGLQSRATTAVSLAEISQNDRGKDALAVRSFPGTVRAK--
          	      . .    .  .   . : .    ::*:.: *:.  .. .    . ..   .   

MG05755.1 	---SPSGYSTPATNIGGLDDAMSVMSFAPTLKPPGDLASLIEGEINRKSRAWQLLRSQSA
NCU09220.1	PQSSASGYTSPRT---DLVETVSVASYPATLRPPGDLADLVTGEFNRRSRAWSMLRTQSS
FG07083.1 	--ASPNRHDSSTS--AEVDETASLMSFAPTMRPAGDIASLLAGELHKKSPAWKMLRTQSA
AN2093.1  	--ASLSHLATPKDG-SDAGDSASVTSYVPYSE-SGDVENIFG---SLASSEVGIAQEEST
          	  :* .   :.  . .   :: *: *: .  . .**: .:.       *    : : :*:

MG05755.1 	SVQPFEARGPNFARPMDQLTGFEQEFDDVNSAAP-------------ADSETGSDDEERL
NCU09220.1	SVQPFEASR---TGACDSLTGFEKEFDNIPELG-----------------EKGITDEQRL
FG07083.1 	SVLPFEKGR---IGADDRFVEFYREFDPLPEEY------------------DEDAEEDVL
AN2093.1  	GLMQFPEFQ-----ADDVEDDFASELEPVGEIGEGGENEGTVLAYGSTTAETDQLIDLVL
          	.:  *         . *    *  *:: : .   ......:  : .:: :.     :  *

MG05755.1 	RLWKAKLKHYMILSSAGKPIWSRHGDLSLINSYMGVVQTIISFYEGAKDPLQGFTAGDAR
NCU09220.1	SLWKSKMKHYMILSSAGKPIWSRHGDTSLINSYMGVVQTIISFYEGAKDPLLGFTAGNAR
FG07083.1 	RLWKSKLKHYLILSSAGKPIWSRYGDLSLINSSMGVVQTIISFYEGAKNPLQGFSAGDTR
AN2093.1  	EKWKAKRKHYIILSAAGKPIWTRHGDGGLISTYVGIIQTIISSYEDSNDRLNGFTAGDTK
          	  **:* ***:***:******:*:** .**.: :*::***** **.::: * **:**:::

MG05755.1 	FVICTQGPLYFVAISRLGESDLQLRAQLDALYMQILSTLTLPTLTNIFVNRPSSDLRKPL
NCU09220.1	FVISTQGPLYFVAISRLGESDAQLRSQLDALYMQILSTLTLPTLKNIFVHRPSTDLRKPL
FG07083.1 	FVILTKGPLYFVAISKLGESDSQIQAQLDALYMQILSTLTLPRLTHIFANRPSTDLRKPL
AN2093.1  	FTVVAKGPLYLVAISRILESDTQLKLQLEALYMQILSTLTLPALTHLFSVRPSTDLKRPL
          	*.: ::****:****:: *** *:: **:************* *.::*  ***:**::**

MG05755.1 	QGTESLLSALADSFTKGSPSAMLGALECLKLRKSHRRAINNAFLKQRSEKLLYGLIVAGG
NCU09220.1	EGTESLLSSLADSFTKGSPSALLGALECLRLRKSHRATINNAFLKCRSDKLLYGLIVAGG
FG07083.1 	EGTEGLLSSLADTFTKGSSSALLGSLECLRLRKSQRHAINNTFLKLRAEKLLYGLIVAGG
AN2093.1  	QGSETLLSTLADSFTKGSPSTLLSALECLKIRKSHRQTINNTLLKTKVSKLLYGLVVAGG
          	:*:* ***:***:*****.*::*.:****::***:* :***::** : .******:****

MG05755.1 	KLVSVIRPRRHSLHPSDLELIFNMLFESDGIKAGGGENWIPLCLPAFNNRGYLYM~YVSF
NCU09220.1	KLVSVIRPRKHSLHPSDLQLIFNMLFESGGIRAGGGENWVPLCLPAFNNRGYLYM~YVSF
FG07083.1 	KLVSVIRPRRHSLHPSDLQLIFNMLFESDGIKGGGGENWIPICLPAFNNKGYLYI2SP--
AN2093.1  	RLVSVVRPKKHSLHPGDLQLLFNMIFEAEAVKAGGGESWIPVCLPGFNSSGYLYI2FF--
          	:****:**::*****.**:*:***:**: .::.****.*:*:***.**. ****:     

MG05755.1 	FD------NKDLDSTSVPEAASPPP------SAQDEIAIILISPDKESFFELKDMRDRVA
NCU09220.1	FDSVETAEENNSNNTNNPEQPPQPPPPKPVTSPDEEIAIILISADKESFFELKSMRDKLA
FG07083.1 	-------------------AAAQGR------SSDEEIAMILVSPDRESFFELKQMRDNVA
AN2093.1  	--------------------------------------------------ELQAMKNALV
          	                                                  **: *:: :.

MG05755.1 	E~TLTKNGALTLIRAATAAGRPRVDAVAGP----------AGGGQIAHFLYKSRANVQLV
NCU09220.1	L~QLAKNGSLALIQSAARQGRPRIETILNTKPLSKEAGQGQGQGQLSHFLYKSRANVQFC
FG07083.1 	Q~QLAKNGTLAIIQNATRVGRPKIHEIAPG-------------SQVSHFLYKSRANVQFC
AN2093.1  	E0KLEENGSINNIKEAIDKGRPSTTDIVPG-------------SVLHHFVYKSRVNVQFI
          	   * :**::  *: *   ***    :                . : **:****.***: 

MG05755.1 	ASSFD----------------------KPRFDMVTRRR~LMTLYHGLHTAVHARYAHSKV
NCU09220.1	QSSLSPAFETSSPPLPSSSPSENGSSQKTTEKLVSRRR~LMTLYHHLHASIHAKHSHLKV
FG07083.1 	MASLEP----------------------HFTGLVSRRR~LMTTYHELHASIHAKHSHLKV
AN2093.1  	MSAYDP----------------------EFSTITRRRR2LISTYNNLHASVHAKHTHVKV
          	 :: ..                          :. *** *:: *: **:::**:::* **

MG05755.1 	LHRAAADSVSLAWITPAFEFYCVAGPNASRAVMTQGANKIVQWIKREEERLFIVGGG~VF
NCU09220.1	LHLVSEDAASLAWITPVFEFYCVAGPNMSSGIMTQCANKVVQWAKREEERLFIIGGG~VF
FG07083.1 	LHGVGDDATSLAWTTPVFEFYCVAGPNVSRATITQGANKIIQWAKREEERLFIIGGG~VF
AN2093.1  	HHCVTQSSSSFAWITPVFELYCVAGPHANRNALAQSASKVVQWVQKEEERLFIISGA0VF
          	 * .  .: *:** **.**:******: .   ::* *.*::** ::*******:.*. **

NCU09220.1	
MG05755.1 	
FG07083.1 	
AN2093.1  	
          	
```
